# Examining Women’s support for birth encouragement policies in China: an extension of the influence of presumed media influence model

**DOI:** 10.3389/fpsyg.2024.1391254

**Published:** 2024-12-06

**Authors:** Shiyao Li

**Affiliations:** Department of Communications and New Media, National University of Singapore, Singapore, Singapore

**Keywords:** influence of presumed influence, support for birth encouragement policies, interpersonal communication, media attention, social norms

## Abstract

This study employs the influence of the presumed media influence (IPMI) model to explore how media messages and interpersonal communication indirectly affect Chinese women’s support for birth encouragement policies. Surveying 616 Chinese women of reproductive age, this study finds that exposure to media messages regarding childbirth benefits and interpersonal communication are positively correlated with the presumed influence of such media messages on others. Presumed media influence on others is further positively associated with perceived personal norms and social norms regarding support for birth encouragement policies. Perceived social and personal norms, in turn, are positively associated with Chinese women’s support for birth encouragement policies. The theoretical and practical implications of this study are examined.

## Introduction

Population growth is crucial for the sustainable development of a country. However, numerous countries are grappling with significant demographic challenges due to aging populations and declining birth rates (Min et al., 2023; Mu, 2019). In 2023, China’s birth rate dropped by 5.7%, reaching a new low (Master, 2024). The low birth rate has negatively impacted Chinese society, leading to a shrinking labor force and adversely affecting economic growth (Mu, 2019). To mitigate the negative impacts of low birth rates, the Chinese government has implemented various birth encouragement policies, such as the two-child policy in 2015 and the three-child policy in 2021. Despite these policy encouragements, the birth rate in China has continued to decline, and Chinese people’s plans to have children remain low, without showing support for these policies (Zhai and Jin, 2023; Zhuang et al., 2020). Billingsley and Ferrarini (2014) indicated that support for the birth policies is positively related to intentions to have children, which will, in turn, affect behavior. Given the importance of support for Chinese birth policies in influencing women’s intentions to have children and subsequent behaviors, understanding how Chinese people react toward the birth encouragement policies (e.g., people’s support for such policies) is of great importance.

The development of the Internet and social media platforms in China has greatly altered the ways people access and process information. Indeed, social media platforms in China have become key arenas for public discourse, shaping opinions on birth policies (Li and Li, 2021; Ning et al., 2022). Past studies have examined the link between media consumption and individuals’ intentions regarding childbirth. For example, Huang and Qiu (2022) found that positive parenting messages on social media can enhance individuals’ plans to have children. Another study by Hornik and McAnany (2001) indicated that media might produce a long-term influence on the intention of having children through media and initiating interpersonal communication among individuals. However, few studies (e.g., Barber and Axinn, 2004) have examined the role of media exposure in shaping people’s attitudes toward having children or its related policies. Given the potential role that media play in shaping public opinions toward childbirth and its related policies (Ma, 2023) and the lack of relevant studies in the context of China, this study aims to understand how media influences Chinese women’s attitudinal reactions (i.e., support) toward birth encouragement policies in China.

Previous studies primarily focused on the direct media influence on individuals’ support for having children, with most of them emphasizing the negative impact of messages highlighting the risks of having children (Marshall et al., 2021; Wan and Zhou, 2023; Yang and Wang, 2023). The majority of these literatures regarded social media platforms as significant channels for disseminating risks (Li et al., 2023; Liu and Song, 2022), contributing to the public’s adverse perception of having children (Li et al., 2023; Mu, 2019; Xu and Hu, 2023; Zhai and Li, 2023). However, on Chinese social media platforms, there is also a substantial amount of content related to the benefits of having children. These messages originate from both the social media accounts of mainstream media and user-generated content (Qian et al., 2020). These contents not only provide the latest updates regarding birth encouragement policies but also disseminate benefit messages related to childbirth, such as the social value, inheritance value, self-development value, and emotional value of having children (Jing et al., 2022). While some studies have indicated the direct influence of media messages about risks on people’s attitudinal responses toward having children (Adair et al., 2014; Barber and Axinn, 2004; Hornik and McAnany, 2001; Knobloch-Westerwick et al., 2016), scant research has explored how media messages on the benefits of having children indirectly affect people’s opinions toward related policies through their presumed influence of on others.

Simultaneously, the topics of having children is frequently discussed within interpersonal communication scenarios in China (Li et al., 2023; Ning et al., 2022). Previous studies have revealed the significant impact of interpersonal communication on shaping attitudinal responses toward childbearing, indicating that personal social networks play a pivotal role in individuals’ decisions about having children (Bühler and Fratczak, 2007; Martins et al., 2013). Boulay and Valente (1999) demonstrated that women usually initiate informal discussions about having children within their social networks, where they gain information and external pressure from the norms of the group. However, there has been limited research on how interpersonal communication might influence people’s attitudinal responses toward having children and relevant birth encouragement policies through presumed media influence.

Extant literature has highlighted how social norms in the IPMI influence attitudinal or behavioral responses (e.g., Ho et al., 2022a). However, few studies have explored how personal norms (i.e., a sense of self-obligation) could complement social norms in shaping the public’s support for birth encouragement policies, especially in China, where cultural values (e.g., filial piety and family continuation) significantly shape the attitudinal responses toward childbirth and governmental birth encouragement policies (Bedford and Yeh, 2019; Dong and Xu, 2016). This internalized sense of obligation constitutes personal norms, leading individuals to perceive childbearing not merely as a duty, but as an essential component of their personal value system (Fu et al., 2020), which subsequently shapes women’s attitudinal and behavioral responses. For example, in the context of birth encouragement policies, support for these policies can be seen as an attitudinal outcome of this internalization of personal responsibilities for childbirth. Therefore, this study seeks to examine the influence of both social and personal norms on support for birth encouragement policies, analyzing how these norms shape individual perspectives.

This study extends the IPMI model by integrating interpersonal communication and personal norms into one theoretical framework, offering new insights into how media messages, interpersonal communication, and normative factors shape the attitudinal response of productive-age women toward birth encouragement policies in China. These findings provide valuable guidance for communication practitioners and policymakers, suggesting that emphasizing interpersonal communication and personal norms could significantly enhance public support for national demographic strategies.

## Literature review

The influence of presumed media influence (IPMI) model extends from the third-person effect, a theory posited by Davison (1983), which suggests that individuals believe others are more susceptible to media influence than they are themselves. At the same time, individuals may adjust their own attitudinal or behavioral responses based on these assumptions (Gunther and Storey, 2003). Gunther (1998) extended the third-person effect by introducing the concept of persuasive inference, explaining how individuals form assumptions about the effects of mass media content on others after encountering it. In the digital media era, people can easily access various media content and form their own impressions. However, despite the abundance of available media content, various algorithm-based media platforms (e.g., AI-driven social media like TikTok) often expose individuals to a limited subset of information that aligns with their interests or preferences, resulting in a narrow window of exposure (Ou et al., 2024; Zuiderveen Borgesius et al., 2016). As a result, individuals tend to be exposed to biased information. This fragmented media exposure leads individuals to form assumptions based on the small sample of content they have encountered, rather than considering the full spectrum of available information (Broniarczyk and Alba, 1994; Gunther, 1998). According to the law-of-small-numbers bias, people tend to make strong inferences based on limited data, assuming that others are exposed to the same content as themselves (Tversky and Kahneman, 1971). According to Davison (1983), individuals tend to perceive others as vulnerable audiences who lack resistance to mass media content, making them more susceptible to its influence. These assumptions can ultimately lead to changes in people’s attitudinal responses, driven primarily by their perception of how others are influenced by the media (Gunther and Storey, 2003).

Specifically, the IPMI suggests that individuals’ attention to media messages leads them to assume that others will also pay attention to the same content and be influenced by it. As a result, they typically adjust their own attitudinal and behavioral response based on these assumptions (Gunther and Storey, 2003). Tal-or and Tsfati (2020) further explained the causal relationship between presumed influence on others and the behavioral component. According to them, there are three main types of presumed influence on others: prevention, coordination, and normative influences. Prevention refers to people’s intention to prevent the further dissemination of harmful messages. As an example, Wu (2023) found that individuals perceiving the harmful influence of health misinformation on others are likely to fact-check the content before sharing it to limit further dissemination. Coordination highlights that based on the presumed influence of an already disseminated message on others, people will adjust their behaviors to align with their assumptions of how the message will influence others (Shi et al., 2022). For instance, Tal-or et al. (2010) found that when individuals are exposed to the positive message of pornography and presume that the same message will influence others, they will weaken their support for the censorship of pornography. Normative influence emphasizes that individuals may actively accept or defy established social norms in response to the presumed effect of the message on others (Shi et al., 2022; Tal-Or and Tsfati, 2020). Previous studies, such as that by Ho et al. (2014), found that perceived prevalence (i.e., descriptive norms) and the perception of other’s expectations (i.e., injunctive norms) regarding drinking influence adolescents’ intention to consume alcohol. The correlation between presumed media influence and behavioral intention has also been demonstrated in previous studies, such as the research on sexual behavioral intentions (Chia, 2006) and that on intentions to consume cultivated meat (Ho et al., 2024). Besides behavioral intentions, the IPMI model has also been found to influence people’s support for certain policies. For example, Ho et al. (2022b) revealed the positive correlation between scientists’ perceived impact of fake science news on others and their support for media literacy education and legislation against fake news. Baek et al. (2019) also found the correlation between fake news exposure and support for government intervention and sanctions against fake news creators and sharers.

The IPMI model proposes two stages in which media indirectly affect attitudinal and behavioral responses. In the first stage, based on the persuasive inference hypothesis, individuals assume that the media messages they are exposed to will also be disseminated to others, leading them to believe that others will attend to and be influenced by these messages (Gunther, 1998; Gunther and Storey, 2003; Ho et al., 2016). Various studies have already demonstrated a positive correlation between self-media attention and presumed media influence on others. For example, Ho et al. (2022a) identified a positive correlation between individuals’ attention to benefit messages regarding plant-based meat and their perception of others’ attention to the same messages. Other studies have also revealed that an individual’s increased exposure to specific media messages is likely to induce their assumptions that others are similarly attentive to those messages. Such media messages include idealized thin images (Park, 2005), fake science news (Ho et al., 2022b), sex-related messages (Chia, 2006) and smoking-related messages (Gunther et al., 2006). Hence, I propose:

*H1*: Self-attention to the benefits of having children on social media will be positively related to their presumed influence of such messages on others.

Besides self-media attention, this study also hypothesizes that interpersonal communication will affect individuals’ presumed media influence on others. In today’s mediatized society, mass communication and interpersonal communication cannot be simply defined in a dichotomous manner (Shi et al., 2022). Southwell and Yzer (2007) revealed in their study that interpersonal communication can play multiple roles in the mass communication process, including being an outcome, mediator, or moderator. People do not receive information in isolation but engage in discussions about specific social content while being exposed to social media (Shi et al., 2022). Furthermore, interpersonal communication is seen as another source of information, through which people actively seek and receive information. In other words, when discussing certain media content, people may believe that such content is likely to influence others, particularly if they assume that these messages are being discussed within their social circles (Chia, 2010; Shi et al., 2022).

Previous studies have focused on the relationships among self-media exposure, interpersonal communication, and presumed media influence. Chia (2010) examined the mediating role of interpersonal communication between one’s own attention to media content and presumed media influence on others. The study found that adolescents’ attention to advertisements was directly and positively related to the frequency with which they discussed these ads with their parents and friends, and this frequency of communication was further positively associated with presumed materialism in others (Chia, 2010). Building on this, Shi et al. (2022) introduced interpersonal communication as a potential variable in the IPMI model and indicated that interpersonal communication has a positive correlation with the presumed influence of COVID-19 misinformation on others.

On various social media platforms in China, there is an abundance of content promoting the benefits of having children, including posts from government accounts and user-generated content. The Chinese government actively utilizes platforms such as WeChat, Weibo, and Douyin (the Chinese version of TikTok) to raise awareness about the advantages of having children and related supportive policies. Key strategies include promoting the three-child policy, emphasizing socio-economic benefits, supporting children’s education, and improving healthcare services for mothers and children (e.g., Jing et al., 2022). In addition to government efforts, celebrities and social media influencers also contribute by promoting positive family values and the joys of parenthood (Qian et al., 2020). They share personal stories and create content that portrays childbearing as emotionally fulfilling and essential for preserving Chinese traditions (e.g., Qian et al., 2020). These messages effectively reach younger audiences, who are highly engaged on platforms like Douyin and Weibo. The content promoting the benefits of having children potentially sparks offline discussions, leading people to assume that others are engaging with these topics as well and may be influenced by them. Therefore, I propose:

*H2*: Individuals’ interpersonal communication about the benefits of having children will be positively related to their presumed influence of messages about the benefits of having children on others.

Social norms include explicit or implicit regulations that guide, regulate, and prescribe social behavior in specific social settings (Burchell et al., 2013). As unwritten rules, they guide people’s actions according to the common and acceptable behaviors of others (Borg, 2022; Tankard and Paluck, 2016). According to Cialdini et al. (1990), social norms can be categorized as descriptive and injunctive norms. Descriptive norms refer to the behavior of the majority and injunctive norms refer to what people feel is right or what they ought to do based on their morals and beliefs (Burchell et al., 2013; Cialdini et al., 1990).

**Figure 1:**
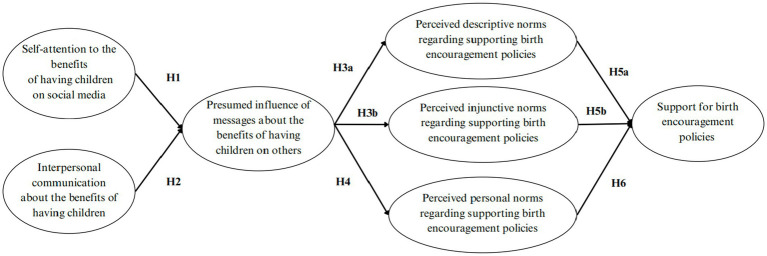
Proposed IPMI model. Oval boxes represent latent variables.

According to Gunther and Storey (2003), when individuals are exposed to certain information, they assume that those around them (e.g., family and friends) have also encountered the same message, leading to a presumed media influence on others. In this process, individuals often employ simple cognitive strategies, believing that media messages affect a large number of people and that these influences are reflected in others’ attitudes and behaviors (Ho et al., 2014). Social norms represent societal expectations or external standards regarding a particular action (Cialdini et al., 1990; Ho et al., 2016). When individuals perceive that the media influences others, they may assume that the attitudes and behaviors promoted by the media will become more prevalent and socially accepted, which in turn shapes their own perceptions of social norms. Previous studies have revealed positive associations between individuals’ presumed media influence and their perceived social norms, including perceived description and injunctive norms. For example, Ho et al. (2022a) found positive relationships between presumed others’ attention to benefit messages on plant-based meat and perceived injunctive norms regarding plant-based meat consumption. Therefore, it is expected that when people perceive the media’s impact on others, they will then perceive others’ approval of the behavior and the social commonality of the behavior, which will further affect behavioral intentions. The proposed relationship is displayed in Figure 1. Hence, I propose:

H3: Individuals’ presumed influence of messages about the benefits of having children on others will be positively related to their (a) perceived descriptive norms and (b) injunctive norms about others’ support for birth encouragement policies.

Personal norms are internalized self-expectations and a sense of moral obligation that guides behavior in specific contexts (Ho et al., 2016; Schwartz, 1977; Thøgersen, 2006). The key characteristic of personal norms is internalization, which Schwartz (1977) describes as the process by which expectations, obligations, and sanctions become embedded within an individual (Bamberg et al., 2007). These norms heighten individuals’ awareness of the consequences of their actions and use moral emotions—such as guilt, shame, and responsibility—to motivate behavior (Thøgersen, 2006). Ho et al. (2016) noted that personal norms offer an alternative mechanism for understanding presumed influence within the IPMI model, as they are internally driven, contrasting with the external foundation of social norms (Cialdini et al., 1990). Their research showed that personal norms can explain the indirect influence of media messages on behavior through moral responsibility and intrinsic motivation, especially in contexts with a moral dimension (Ho et al., 2016). In China, the culture of childbearing can be summarized as “more children, more blessings,” a traditional belief that has endured for thousands of years and has been integrated into social norms, thereby influencing individuals’ decisions to have children (Bedford and Yeh, 2019). Media content promoting the benefits of having children frequently highlights positive values such as self-fulfillment, filial piety, and the preservation of Chinese traditions, portraying them as virtues that align with cultural ideals (Bedford and Yeh, 2019; Qian et al., 2020; Yu and Liang, 2022). These contents emphasized the importance of childbearing for individuals, families, and society, framing it as a means of meeting societal expectations and fulfilling moral obligations (Dong and Xu, 2016; Bedford and Yeh, 2019). Hence, supporting birth encouragement policies is therefore not merely an action aligned with social expectations, but also a way for individuals, particularly women, to fulfill responsibilities to family and society. Hence, I propose:

H4: The presumed influence of messages about the benefits of having children on others will be positively related to perceived personal norms regarding support for birth encouragement policies.

Over the past few years, China has adjusted its birth policies to counteract the adverse effects of a low birth rate on society and encourage people to have children (Zhai and Jin, 2023). These policies include loosening restrictions on the number of children, subsidizing the costs of having children, encouraging labor participation, and promoting gender equality (Billingsley and Ferrarini, 2014; Gauthier and Philipov, 2008). Zhuang et al. (2020) argued that despite the continuous implementation of new policies, Chinese people’s intention of having children remains low, and they have not shown support for these policies. In the study of family policy and intention to have children, Billingsley and Ferrarini (2014) indicated that support for family policies is positively related to the intention of having children, which will in turn affect behavior. Given the importance of support for Chinese birth policies in affecting women’s intentions and behaviors regarding having children, this study aims to examine what will affect Chinese women’s support for Chinese birth policies.

Although both are components of social norms, descriptive and injunctive norms serve distinct functions in predicting and influencing behaviors: descriptive norms provide information about common actions, while injunctive norms dictate societal expectations of approval or disapproval (Cialdini et al., 1990; Paek, 2009). Given their different natures, this study investigates descriptive and injunctive norms separately and examines their respective roles in the IPMI. Previous research has found that both perceived descriptive and injunctive norms were positively related to individuals’ attitudinal responses across various contexts (e.g., Ho et al., 2022a). For instance, Ho et al. (2014) discovered a positive correlation between both descriptive and injunctive norms and the favorable perception of alcohol consumption. Similarly, Ho et al. (2024) identified a positive link between the perception of these norms and the intention to consume cultivated meat. However, little research has focused on the relationship between these two types of norms and public support for birth encouragement policies in the context of China. Therefore, I propose:

H5: Individuals’ (a) perceived descriptive norms and (b) perceived injunctive norms regarding others’ support for birth encouragement policies will be positively associated with their own support for birth encouragement policies.

The IPMI model suggests that individuals often perceive media content to have effects on others, and thereby adjust their attitudinal and behavioral responses (Gunther and Storey, 2003; Tal-or et al., 2010). After the Chinese government launched the birth encouragement policy, many scholars noticed how China is using government social media accounts to strengthen people’s awareness of the risks of low birth rate (e.g., labor shortage, imbalanced population structure, decline in innovation) and the benefits of birth encouragement policies (Jing et al., 2022). According to Davison (1983), when media promotes negative behavior, individuals infer that the behavior will also have negative impacts on others. This adverse impact will stimulate people’s awareness of the consequences, leading them to believe that they should assume social responsibility (Ho et al., 2022b). At the same time, the exposure to the benefits of the birth encouragement policies in mass media will also increase people’s awareness of the social rewards of having children, making them support the birth encouragement policies as an alternative to bearing social responsibility (Thøgersen, 2009).

Ho et al. (2022b) indicated that personal norms formed by scientists’ perceptions of the influence of fake science news on others are positively related to support for relevant education and legislation. In the study on public support for green transport policy, Zhang et al. (2020) indicated that personal norms can predict the acceptance of green transport policies. Additionally, Steg et al. (2005) found that there is a positive relationship between pro-environmental personal norms and support for energy policies. In this study, I propose that people’s perceptions of personal norms will have positive effects on their support for China’s birth encouragement policies. The proposed relationship is displayed in Figure 1. Therefore, I hypothesize:

H6: Individuals’ perceived personal norms will be positively related to their own support for birth encouragement policies.

## Methods

### Participants

An online survey was administered to Chinese women aged between 20 and 40, who are above the legal marriage age and within the optimal reproductive age. The survey study was conducted from October to November 2023. The respondents were recruited using the online panel service provided by a reputable market research company in China, i.e., Wenjuanxing.com. A total of 4,941 respondents were invited but only 616 Chinese females completed the whole survey. Respondents who completed the survey were compensated with points by the research company, and the survey had an approximate duration of 20 min.

**Figure 2:**
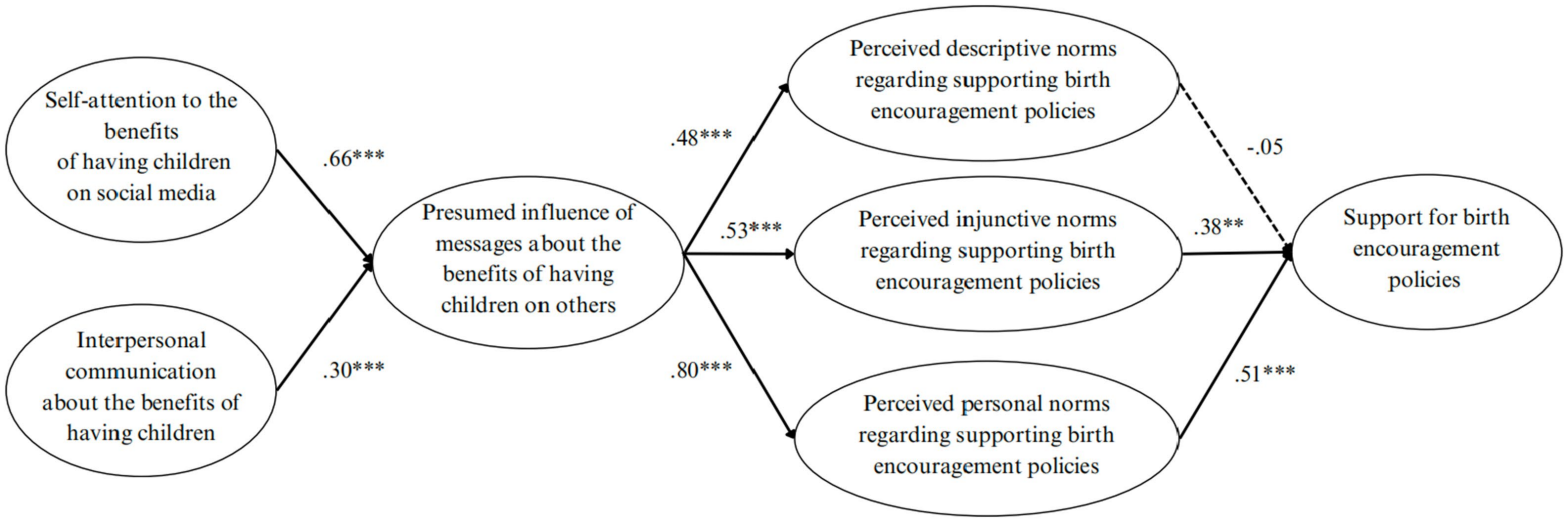
Extended IPMI model. Oval boxes represent latent variables.

All the 616 respondents were aged from 20 to 40 years old (M_age_ = 29; SD_age_ = 4.89). The median education level among the respondents was a bachelor’s degree. The median monthly household income ranges from 6,000 to 8,999 RMB. Within the surveyed respondents, 64.6% were married, 33.6% were single, and 1.7% were divorced. In terms of parenthood, 45.9% had no children, 41.6% had one child, and 12.5% had two or more children (Figure 2).

### Measures

All the measures used in this study were derived from existing research, with slight adjustments made to each measurement to ensure relevance to the specific context of the current study. The measurements and descriptive statistics for all the items are displayed in Table 1. Unless otherwise specified, all variables were evaluated through a 5-point Likert scale, which scaled from 1 (Strongly disagree) to 5 (Strongly agree).

**Table 1:** Factor loadings of all items.

	Items	Factor loading	M	SD	CR	AVE	Citations
Self-attention to media content on the benefits of having children	When using social media, excluding viewing news content and watching TV programmes, how much attention do you pay to messages on:		3.493	0.845	0.764	0.518	Ho et al. (2020) and Liao et al. (2016)
	(a) The social benefits of having children (e.g., mitigating aging issues)	0.744					
	(b) The benefits of having children on family heritage (e.g., passing on familial traditions, values, and accumulated wealth)	0.736					
	(c) The emotional benefits of having children (e.g., the unique joy derived from witnessing the children’s growth)	0.679					
Interpersonal communication about the benefits of having children	(a) How often do you discuss the benefits of having children with your family or relatives?	0.840	3.104	0.840	0.768	0.528	Shi et al. (2022)

	(b) How often do you discuss the benefits of having children with your friends?	0.657					
	(c) How often do you talk to others on social media platforms about the benefits of having children?	0.667					
Presumed influence of messages about the benefits of having children on others	How much influence do you think messages on the benefits of having children have on…		3.179	0.767	0.762	0.517	Ho et al. (2020)
	(a) Your family members	0.725					
	(b) Your close friends	0.736					
	(c) General Public in China	0.694					
Perceived personal norms regarding supporting birth encouragement policies	How much do you agree or disagree with the following statements?		2.867	1.125	0.875	0.701	Harland et al. (1999)
	(a) I have a strong sense of personal responsibility for supporting birth encouragement policies and consider it my duty	0.889					
	(b) I’m willing to go the extra mile to support birth encouragement policies	0.849					
	(c) If I did not support birth encouragement policies, I would probably feel ashamed	0.770					
Perceived descriptive norms regarding supporting birth encouragement policies	How much do you agree or disagree with the following statements?			3.748	0.671	0.531	Ho et al. (2014) and Ho et al. (2020)
	(a) I think my family members would support birth encouragement policies.	0.697					
	(b) I think my close friends would support birth encouragement policies.	0.718					
	(c) I think people in China would support birth encouragement policies.	0.768					
Perceived Injunctive norms regarding supporting birth encouragement policies	How much do you agree or disagree with the following statements?			3.725	0.667	0.508	Ho et al. (2014) and Ho et al. (2020)
	(a) I think my family members would approve of me supporting birth encouragement policies in China.	0.677					
	(b) I think my close friends would approve of me supporting birth encouragement policies in China.	0.773					
	(c) I think people in China would approve of me supporting birth encouragement policies in China.	0.684					
Support for birth encouragement policies	How much do you agree or disagree with the following statements?		3.805		0.843	0.456	Ho et al. (2020)
	(a) I support China’s policies to encourage childbirth (e.g., encourage having two or three children).	0.908					
	(b) I support China’s economic policies that encourage childbirth (e.g., providing maternity allowances)	0.737					

CR, Composite reliability; AVE, Averaged variances extracted.

*Self-attention to media content on the benefits of having children* was assessed through three items, which were modified from those used in prior research (Ho et al., 2020; Liao et al., 2016). Respondents rated the degree of attention they devoted to various birth-related media messages (e.g., social benefits, benefits for family heritage, and emotional benefits) on social media, using a scale from 1 (no attention at all) to 5 (a lot of attention) (M = 3.493, SD = 0.845).

*Interpersonal communication about the benefits of having children* was measured by 3 items adapted from previous study (Shi et al., 2022). Participants were required to assign a rating of 1–5 (1 = not at all; 5 = very frequently), how often they had discussed the benefits of having children with (a) family or relatives, (b) close friends, (c) people on social media platforms (M = 3.104, SD = 0.840).

*Presumed influence of messages about the benefits of having children on others* was assessed through three items that were adapted from Ho et al. (2020). Participants were asked to evaluate on a 1-to-5 scale (1 = not at all; 5 = a lot of influence), how much influence they think messages on the benefits of having children have on each referent group (family members, close friends, general public in China) (M = 3.179, SD = 0.767).”

*Perceived descriptive norms regarding others’ support for birth encouragement policies* were examined through three items adapted from previous research (Ho et al., 2014; Ho et al., 2020). Respondents were asked to rate on a scale of 1 (strongly disagree) to 5 (strongly agree), to what level they agree or disagree that each referent group (a) family members, (b) close friends, (c) people in China will support the birth encouragement policies in China (M = 3.748, SD = 0.671).

*Perceived injunctive norms regarding others’ support for birth encouragement policies* were measured using three items modified from past studies (Ho et al., 2014; Ho et al., 2020). Respondents were asked to rate on a scale of 1 (strongly disagree) to 5 (strongly agree), to what level they agree or disagree that each referent group (a) family members, (b) close friends, (c) people in China approves of them to support the birth encouragement policies in China (M = 3.725, SD = 0.667).

*Perceived personal norms regarding own support for birth encouragement policies* were measured using 3-item modified from previous studies (Harland et al., 1999). On a scale of 1–5 (1 = strongly disagree; 5 = strongly agree), participants were asked to evaluate to what extent they agree with the following items: (a) “I have a strong sense of personal responsibility for supporting birth encouragement policies,” (b) “I am willing to go the extra mile to support for birth encouragement policies,” and (c) “If I did not support for birth encouragement policies, I would probably feel ashamed” (M = 2.867, SD = 1.125).

*Support for birth encouragement policies* were measured using 2 items, including (a) I support China’s policies to encourage having children (e.g., encourage having two or three children), (b) I support China’s economic policies that encourage having children (e.g., providing maternity allowances) (M = 3.805, SD = 0.843).

### Analytical approach

This study used the software *Mplus* to conduct the data analysis. The measurement model was tested using confirmatory factor analysis. Structural equation modeling was employed to test the proposed structural model. I controlled for gender, age, education, number of children, and income in the data analysis. The model fit was evaluated using the following criteria: (a) the maximum likelihood chi-square (*χ*^2^) value should not be significant (*p* > 0.05) (Schermelleh-Engel et al., 2003), (b) the relative chi-square ratio (*χ*^2^/*df*) should not exceed 5 (Wheaton et al., 1977), (c) the root mean square error of approximation (RMSEA) value should be below 0.08 (MacCallum et al., 1996), (d) the standardized root-mean-square residual (SRMR) should be below 0.08, and (e) both the comparative fit index (CFI) and Tucker–Lewis Index (TLI) should exceed 0.95 (Hu and Bentler, 1999). Items with factor loadings below 0.55 were removed to ensure good reliability of the resulting items (Comrey and Lee, 2013).

## Results

All latent variables were defined within the measurement model. The factor loadings of all items exceeded 0.60. Table 1 displays the factor loadings Average Variance Extracted (AVE), and Composite Reliability (CR) of all latent variables included in this study. Confirmatory factor analysis revealed that the measurement model exhibited a good fit, *χ^2^* = 251.99, *df* = 143, *p* < 0.001; *χ^2^/df* = 1.76, CFI = 0.97, TLI = 0.96, RMSEA = 0.04, SRMR = 0.04. The structural equation modeling (SEM) further indicated that the extended IPMI model demonstrated an acceptable fit, *χ^2^* = 436.57, *df* = 171, *p* < 0.001; *χ^2^/df* = 2.55, CFI = 0.95, TLI = 0.95, RMSEA = 0.05, SRMR = 0.06 (see Table 2).

**Table 2:** The model fit indices of measurement model and extended IPMI model (*N* = 616).

Model fit indices	*χ* ^2^	*df*	CFI	TLI	RMSEA	SRMR	*χ*^2^/*df*
Measurement model	251.99	143	0.97	0.96	0.04	0.04	1.76
Structural model	436.57	171	0.95	0.94	0.05	0.06	2.55

H1 hypothesized the relationship between individuals’ own attention to media content about the benefits of having children and their presumed influence of such messages on others. Results indicated that individuals’ own attention to media content about the benefits of having children exhibited positive associations with their presumed influence of such messages on others (*β* = 0.66, *p* < 0.001). Hence, H1 was supported. H2 hypothesized the associations between individuals’ interpersonal communication about the benefits of having children and their presumed influence of childbirth-related messages on others. Results demonstrated that individuals’ interpersonal communication about the benefits of having children was positively related to their presumed influence of such messages on others (*β* = 0.30, *p* < 0.001), supporting H2.

H3 (a) and H3 (b) hypothesized the association between individuals’ presumed influence of messages about the benefits of having children on others and perceived descriptive and injunctive norms regarding others’ support for birth encouragement policies. Results revealed that individuals’ presumed influence of messages about the benefits of having children on others exhibited positive associations with their perceived descriptive norms (*β* = 0.48, *p* < 0.001) and injunctive norms (*β* = 0.53, *p* < 0.001), supporting H3 (a) and H3 (b). Additionally, H4 posited the relationship between individuals’ presumed influence of messages about the benefits of having children on others and their perceived personal norms. The findings showed that individuals’ presumed influence of messages about the benefits of having children on others was positively associated with their perceived personal norms regarding own support for birth encouragement policies (*β* = 0.80, *p* < 0.01), thus supporting H4.

H5 (a) and H5 (b) Hypothesized the association between individuals’ perceived descriptive and injunctive norms regarding others’ support for birth encouragement policies and their own support for policies encouraging childbirth. Results revealed that individuals’ perceived injunctive norms were positively associated with their own support for policies encouraging childbirth (*β* = 0.38, *p* = 0.01), supporting H5 (a). However, the relationship between individuals’ perceived descriptive norms regarding others’ support for birth encouragement policies and their own support for policies encouraging childbirth was non-significant (*β* = −0.05, *p* = 0.73). Hence, H5 (a) was not supported. Additionally, H6 posited the relationship between individuals’ perceived personal norms and their support for policies encouraging childbirth. The findings showed that individuals’ perceived personal norms were positively associated with their own support for policies encouraging childbirth (*β* = 0.51, *p* < 0.001), thus supporting H6. Finally, the structural equation modeling results also showed the relationships between the controlled variables and individuals’ support for policies encouraging childbirth. Specifically, age (*β* = 0.08, *p* = 0.03) was positively related to individuals’ support for policies encouraging childbirth while education (*β* = 0.01, *p* = 0.79) was not significantly related to individuals’ support for policies encouraging childbirth.

## Discussion

Using the IPMI model, this research explores the indirect effects of women’s attention to media messages about the benefits of having children on their support for birth encouragement policies in China. It examines how interpersonal communication shapes perceptions of presumed influence on others and investigates the roles of social and personal norms in shaping support for these policies. The findings of this research support most of the hypotheses, with the exception of the relationship between perceived descriptive norms and support for birth encouragement policies.

### The positive relation between own attention and others’ attention

The IPMI model suggests that individuals base on their own attention to media messages to infer others’ attention to the same content (Eveland et al., 1999; Gunther and Storey, 2003). Consistent with previous studies applying the IPMI model (Ho et al., 2022a; Hong and Kim, 2020), this research also found that personal attention to media messages about the benefits of having children is positively correlated with the presumed influence of such messages on others. Notably, we discovered that interpersonal communication is also positively and directly associated with the presumed influence of these benefit messages on others. This aligns with prior research (Shi et al., 2022), which found that interpersonal communication about COVID-19 was similarly linked to presumed attention to COVID-19-related messages. This finding can be interpreted from two perspectives. First, in China, interpersonal communication plays a crucial role in discussions about having children (Li et al., 2023). These conversations often occur among relatives, friends, and social networks, exerting significant influence on personal decisions and attitudinal responses toward childbearing (Li et al., 2023). Additionally, interpersonal communication serves as a valuable channel for gaining insights into social issues and gauging others’ engagement (Shi et al., 2022). Therefore, it is understandable that interpersonal communication would have a positive impact on the perception of media influence on others.

### The positive relation between presumed influence and normative perceptions

The results indicate a positive relationship between the presumed influence of benefit messages on others and personal norms, which is consistent with previous IPMI studies examining the assumed impact of fake scientific news on others and individual standards for rejecting such misinformation (Ho et al., 2022b). Personal norms are often activated when individuals internalize expectations, obligations, and sanctions that are self-referenced (Schwartz, 1977). Following China’s implementation of policies promoting childbirth, the advantages of increasing birth rates—such as reversing population decline and improving the demographic structure—have been widely disseminated by both government social media accounts and social media influencers (Lin, 2021; Xiong et al., 2016). Women’s exposure to these narratives may lead them to believe that others are similarly shaped by these messages, reinforcing the perception that supporting governmental childbirth encouragement policies is not only socially expected but also a personal responsibility for the country’s sustainable development. In China’s collectivist context, where collective responsibilities often take precedence over individual priorities, individuals are more inclined to see national responsibilities as their own (Tjosvold et al., 2003; Triandis, 1993; Yang et al., 2024). This perspective fosters their stronger support for government-issued policies.

This study also examines the correlation between presumed media influence on others and social norms, finding a positive association between perceived social norms (including both injunctive and descriptive norms) and presumed media influence on others. This finding aligns with earlier research on topics like plant-based meat and nano-enabled food products (Ho et al., 2020; Ho et al., 2022a). For example, Ho et al. (2022a) discussed that due to the public’s limited familiarity with novel foods, individuals tend to rely more on their perceptions of others’ consumption behaviors to estimate the prevalence of these behaviors. Although encouraging childbirth is not a new issue in China, women still rely on their perceptions of others’ media exposure to positive content on this topic to estimate others’ reactions toward birth encouragement policies, subsequently shaping their attitudinal response (e.g., support) toward these policies (Zhang et al., 2021). This finding further confirms that even on a familiar issue like childbirth, people’s attitudinal responses can still be influenced by presumed media influence on others, mediated through normative perceptions—namely, perceptions of most others’ behavioral and attitudinal responses.

The positive association between the presumed influence of childbirth-related messages and social norms can also be explained by the bandwagon effect, wherein individuals perceive attitudinal responses toward childbearing as increasingly favorable and align their attitudinal response with what they interpret as the majority opinion in society (Henshel and Johnston, 1987). Similarly, when women are exposed to positive messages about childbirth, they often assume that others are similarly influenced, reinforcing their perception of social expectations and general acceptance of these messages. This process leads them to conform to these perceived norms to gain social approval. In a collectivist society like China, which highly values social harmony and adherence to group expectations, such influences may be even more pronounced, impacting attitudinal and behavioral responses across various domains.

### The positive relation between normative perceptions and support for policies

The results also indicated that injunctive norms were positively associated with support for birth encouragement policies. One possible explanation for this positive relationship is that Chinese society, as a collectivist culture deeply influenced by Confucian values, emphasizes group conformity, social orientation, and interdependence (Jiang et al., 2019; Wang et al., 2023). In such a context, where the cost of deviating from social expectations is high, individuals are more likely to adhere to collective norms and engage in socially desirable behaviors (Cialdini et al., 1990; Wang et al., 2023; Yin et al., 2018). Furthermore, in China, government policies are often viewed as reflective of the prevailing societal expectations for individual behavior (Du et al., 2022). In a society that places a high value on conformity and alignment with governmental directives, supporting state policies is seen as behavior that aligns with social approval (Zhou and Sun, 2017). The ongoing introduction of favorable birth policies can be interpreted as a societal or governmental signal to promote childbirth. This perception of approval contributes to the positive association between injunctive norms and support for these policies.

Additionally, the findings demonstrate a positive correlation between personal norms and support for birth encouragement policies in China. This aligns with previous research (Zhang et al., 2020), which has shown a significant relationship between personal norms and the adoption of green transportation policies. Ho et al. (2016) argued that personal norms are shaped by the assimilation of media messages, which adjust attitudinal responses by reinforcing a sense of responsibility in alignment with societal expectations. In China, childbearing is viewed as a means of achieving family continuity, fulfilling filial piety, and providing an important path for women’s self-fulfillment (Yu and Liang, 2022). In the context of China’s low birth rate, both media narratives and birth encouragement policies convey personal obligations and social responsibilities that individuals are expected to fulfill (Zhai and Jin, 2023). When individuals learn about the positive impact of childbearing on society and family through media content, this not only makes them aware of societal expectations regarding childbearing but also fosters their recognition of the value of having children, thereby transforming this awareness into an internalized sense of personal obligations (i.e., personal norms; Morris et al., 2015). These internalized personal norms lead individuals to support birth encouragement policies, viewing childbearing as a means of achieving personal value and fulfilling family responsibilities.

### The non-significant relation between descriptive norms and support for policies

While the study found support for most relationships, descriptive norms did not show a significant association with policy support. Descriptive norms are understood as behaviors that are generally considered normal or typical in society (Cialdini et al., 1990; Ho et al., 2022a) The non-significant role of perceived descriptive norms on support for birth encouragement policies indicates that the prevalence of support for birth encouragement policies among social networks is still insufficient for Chinese women to generate same support for such policies. This may be because birth encouragement policies often involve deeply personal decisions influenced by economic realities, such as financial stability, housing costs, and career demands. While individuals may perceive that others support these policies, they often base their own decisions on personal economic assessments and readiness to have children (Brehm, 1966). In China, for instance, high living costs, educational expenses, and housing affordability concerns are frequently cited as barriers to increasing family size, regardless of perceived support for government policies (Li et al., 2022; Wang and Zhou, 2021). Consequently, even perceived widespread support for birth encouragement policies may have limited influence on personal support, as individual choices are shaped by unique personal, economic, and cultural factors that descriptive norms alone may not capture in the non-significant relationship between descriptive norms and support for these policies.

## Theoretical and practical implications

This study offers several contributions from a theoretical perspective. First, I employed the IPMI model in an unexplored context, investigating women’s support for birth encouragement policies in China. This study contributes to the IPMI literature by exploring the roles of interpersonal communication and personal norms in the IPMI model to explain people’s support for birth encouragement policies in China, a topic previously underexplored in research. In line with Shi et al. (2022), this research demonstrates that interpersonal communication significantly influences attitudinal variables (i.e., support for birth encouragement policies) through presumed influence on others. By applying the IPMI model to examine women’s support for birth encouragement policies, this study provides new insights into the relationships among media attention, interpersonal communication, normative factors, and attitudinal responses. It suggests that these elements indirectly shape the attitudinal response toward national demographic strategies.

Practically, this study offers some insights for communication practitioners and the government on disseminating birth policies in China. The results indicate that interpersonal communication positively impacts the perceived influence of media messages about the benefits of having children on others. Therefore, leveraging and enhancing interpersonal communication about the benefits of having children could significantly bolster public support for birth policies. Media professionals might explore innovative methods to simulate conversations. This might include collaborations with social media influencers and the utilization of live-streaming platforms to facilitate dynamic exchanges among individuals. In today’s context, where interpersonal communication includes digital interactions, strategies could focus on social media engagement and collaborating with key opinion leaders (KOLs) to initiate dialogues about having children.

In light of the study’s findings, future campaigns to promote having children should place a greater emphasis on personal norms rather than descriptive norms. Media narratives should pivot toward invoking a sense of personal duty and moral obligation, aiming to resonate with women’s intrinsic values. When crafting messages, media professionals are encouraged to incorporate moral elements that strike a chord with core cultural values, such as filial piety and social responsibility, to address the population issue. Moreover, plans to have children could be positioned as a commitment that women are expected to fulfill, aligning with familial and societal anticipations. This framing could be more effective if it taps into the cultural narrative of contributing to the larger community, thereby presenting having children not just as a personal choice, but as a contribution to societal continuity.

## Limitations and future studies

Firstly, this study employed a cross-sectional survey as a methodological approach to investigate the relationships between variables. Future research could employ experimental methodologies to delineate the causal linkages within the IPMI framework. Secondly, the sample was restricted to Chinese women aged 20 to 40, which provided insight primarily into the perspectives of women of reproductive age. However, support for birth encouragement policies or plans to have children are not solely determined by women and family planning decisions are often made jointly by couples. Therefore, subsequent studies could expand the demographic range to include males, as well as diverse ages, locations, ethnicities, and educational levels (Xu and Hu, 2023; Zhang, 2022). Thirdly, the current research did not explore additional factors that could be integrated into the IPMI model, factors that may influence women’s support for birth encouragement policies. These include individuals’ responsiveness to risk messages associated with having children and the specific mediums (such as online platforms or face-to-face interactions) where interpersonal communication takes place. Future research endeavors could incorporate these elements to enhance the IPMI model’s comprehensiveness and explanatory power.

## Conclusion

China is currently facing significant population problems and a dropping birth rate, despite the implementation of birth encouragement policies, the situation continues to deteriorate. Researchers have not yet fully understood how women’s media attention to the messages highlighting the benefits of having children indirectly influences their support for birth encouragement policies. Drawing on the IPMI model, this study suggests that interpersonal communication, together with self-media attention, plays significant roles in influencing women’s presumed influence of benefit messages about having children on others and their normative perceptions, which further influence their support for birth encouragement policies. This study provides valuable insights and potential suggestions for media practitioners and the government to communicate birth encouragement policies to the public in China.

## Data Availability

The original contributions presented in the study are included in the article/supplementary material, further inquiries can be directed to the corresponding author.
